# Frequency and Characteristics of Myelin Oligodendrocyte Glycoprotein Antibody‐Associated Disease Accompanied by Fever: A Single‐Center Retrospective Study

**DOI:** 10.1002/cns.71153

**Published:** 2026-09-15

**Authors:** Ai Guo, Fu‐Dong Shi, Kaibin Shi

**Affiliations:** ^1^ Department of Neurology, Beijing Tiantan Hospital Capital Medical University Beijing China; ^2^ Department of Neurology Tianjin Medical University General Hospital Tianjin China; ^3^ Chinese Institute for Immunology Chinese Institutes for Medical Research Beijing China; ^4^ Beijing Key Laboratory of Autoimmune Disease Mechanism Research and Novel Drug Development Beijing China

**Keywords:** cerebral cortical encephalitis, cerebrospinal fluid pleocytosis, fever, myelin oligodendrocyte glycoprotein antibody‐associated disease (MOGAD)

## Abstract

**Aims:**

To evaluate the frequency and characteristics of myelin oligodendrocyte glycoprotein antibody‐associated disease (MOGAD) accompanied by fever.

**Methods:**

This retrospective study was conducted from April 2019 to December 2025 at Beijing Tiantan Hospital in China. Patients who met the 2023 international MOGAD panel diagnostic criteria and had a disease duration of at least 6 months were included. Febrile and afebrile groups were compared using Mann–Whitney *U*, *χ* ^2^, and log‐rank tests pre‐ and post‐propensity score matching. Logistic regression identified factors associated with fever.

**Results:**

A total of 123 patients were included. Fever occurred during 43 acute attacks in 38 (30.9%) patients, with the majority (33 attacks) representing the initial episode. The median maximal temperature was 38.6°C. Cerebral cortical encephalitis was the leading phenotype in febrile attacks (48.8%). Compared to the afebrile group, the febrile group exhibited a significantly higher median cerebrospinal fluid (CSF) leukocyte count (56/μL vs. 11.5/μL; *p* < 0.001) and CSF protein (52.65 mg/dL vs. 39.76 mg/dL; *p* = 0.001). Cerebral cortical encephalitis and CSF pleocytosis were independently associated with fever in the multivariable model.

**Conclusions:**

Fever was relatively common in our MOGAD cohort, occurring in approximately one‐third of patients and across all clinical phenotypes. Both cerebral cortical encephalitis and CSF pleocytosis were independently associated with fever.

## Graphical abstract

Diagnostic pitfall in fever with CNS involvement: MOGAD. Fever accompanied by neurological symptoms was initially suspected as central nervous system infection. However, negative pathogen tests and failed anti‐infective therapy, followed by positive MOG‐IgG, led to a revised diagnosis of MOGAD. The patient responded favorably to corticosteroids or IVIG. Notably, MOGAD can mimic infection with fever at disease onset, and fever may be a manifestation of MOGAD itself.

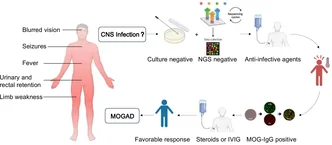

## 1 Introduction

Myelin oligodendrocyte glycoprotein antibody‐associated disease (MOGAD) is a rare autoimmune inflammatory disorder of the central nervous system (CNS) driven by autoantibodies against myelin oligodendrocyte glycoprotein (MOG), a type I integral membrane protein localized to CNS myelin sheaths [1, 2]. Core clinical phenotypes of MOGAD include optic neuritis, myelitis, acute disseminated encephalomyelitis (ADEM), cerebral monofocal or polyfocal deficits, brainstem or cerebellar deficits, and cerebral cortical encephalitis [3]. While fever frequently accompanies disease activity in autoimmune rheumatic diseases such as systemic lupus erythematosus (SLE), it is an uncommon feature in nervous system autoimmune disorders, exemplified by multiple sclerosis (MS), where its presence is regarded as a red flag, often prompting suspicion of an infectious rather than an autoimmune etiology [4, 5, 6, 7, 8]. Recently, fever has been increasingly mentioned in the context of MOGAD, typically as a concomitant or prodromal symptom [9, 10, 11]. A review of case reports identified fever in 39% of cases [7]. Vrajesh et al. further described prolonged fever in 12 pediatric patients [12]. Despite this growing recognition, the frequency and clinical characteristics of fever in MOGAD have not been systematically examined in a large cohort. This knowledge gap may contribute to the persistent under‐recognition of fever as a potential MOGAD manifestation in clinical practice, potentially delaying diagnosis and appropriate treatment [6, 12]. This study aimed to systematically characterize the frequency and clinical features of fever in a MOGAD cohort, with the goal of helping clinicians recognize fever as a potential manifestation of MOGAD rather than an automatic exclusion criterion.

## 2 Methods

### 2.1 Study Design and Participants

This retrospective cohort study included patients with MOGAD at Beijing Tiantan Hospital from April 2019 to December 2025. All patients provided written informed consent. We included all consecutive patients who fulfilled the 2023 international MOGAD diagnostic criteria and had a disease duration of at least 6 months. And MOG‐IgG positivity was determined via a cell‐based assay (CBA). The follow‐up included outpatient, inpatient, and telephone follow‐ups. We followed the STROBE reporting guidelines.

### 2.2 Clinical Data Collection

Data were collected from electronic medical records. Patients were classified into the febrile group if they met all the following criteria: (1) armpit temperature of 37.3°C or higher. (2) fever occurred within a time window from 3 day before to 7 days after the onset of neurological symptoms during an acute attack. Serum samples with MOG‐IgG titers of 1:100 or higher were considered clearly positive. Lumbar puncture data were obtained from the earliest available acute attack in the afebrile group and from the earliest febrile attack in the febrile group. Raised cerebrospinal fluid (CSF) opening pressure (OP) is defined by 250 mmH_2_O or higher [13]. CSF pleocytosis was defined as leukocyte > 50 cells/μL [14]. IL‐6 concentrations in peripheral blood and CSF were measured by chemiluminescence assay at our hospital's clinical laboratory, where the normal reference range was 2–3.4 pg/mL. Levels below the lower limit of detection (2 pg/mL) were assigned a value of 2 pg/mL, and levels exceeding 10 times the upper limit of normal (i.e., > 34 pg/mL) were defined as elevated. Recurrence status was established using medical records and MRI findings. All data were deidentified to ensure patient anonymity.

### 2.3 Statistical Analysis

Data processing and analysis were performed using graphpad 9.0, R version 4.3.3 (2024‐02‐29), along with Zstats 1.0 (www.zstats.net). The normality of the data was assessed using the Shapiro–Wilk test. Quantitative variables with a normal distribution were expressed as mean ± standard deviation (SD), while those without a normal distribution were presented as median (interquartile range [IQR]). Categorical variables were reported as frequencies and percentages. The proportion of available data for each variable is reported in the baseline table, and all analyses were performed using available data. To compare demographic, clinical, and laboratory characteristics between the two groups, the Mann–Whitney *U* test, and *χ* ^2^ test were used as appropriate. A sensitivity analysis was performed for body temperature cut off of 38.1°C or higher. To find factors associated with fever, univariate and multivariate logistic regression analyses were performed. Variables with a *p* value < 0.20 in the univariate analysis were entered into the multivariate model. To balance important patient characteristics between groups, a 1:1 propensity score matching (PSM) was performed using the nearest‐neighbor matching algorithm with a caliper of 0.2. Then, age at onset, gender, onset phenotype, EDSS score, serum MOG‐IgG titer, onset immunosuppressive therapy was used as a covariate for PSM. Covariate balance was evaluated using standardized mean differences (SMDs), where an absolute SMD of less than 0.2 was taken as evidence of acceptable balance. Time to first relapse was analyzed using Kaplan–Meier (K–M) curves, with comparisons between febrile and afebrile groups performed using the log‐rank test. All tests were two‐sided, and a *p* < 0.05 was considered statistically significant.

## 3 Results

### 3.1 Proportion of MOGAD Patients Presenting With Fever During Acute Episodes

**FIGURE 1:**
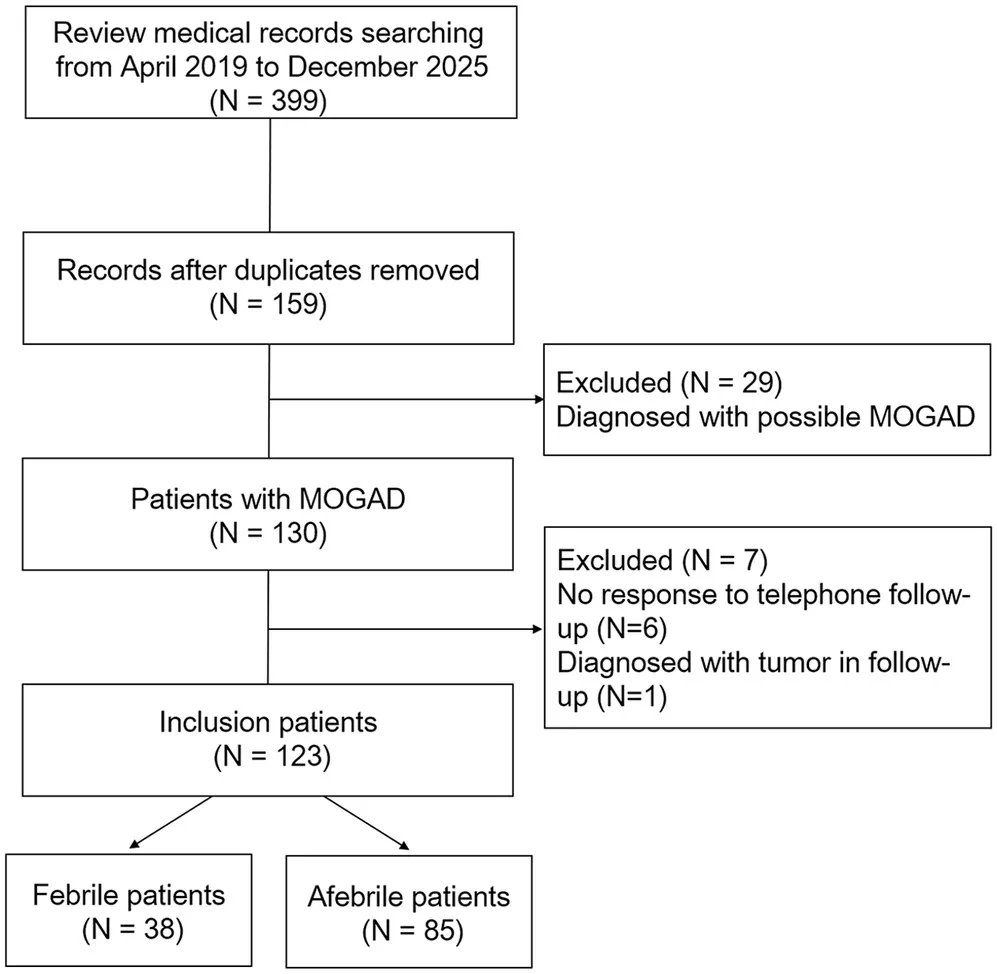
Flowchart illustrating the patient selection process and study enrollment. MOG, myelin oligodendrocyte glycoprotein; MOGAD, myelin oligodendrocyte glycoprotein antibody‐associated disease.

A total of 123 patients were included (Figure 1). Fever accompanied by core symptoms occurred in 38/123 (30.9%) MOGAD patients. Demographic and clinical characteristics before PSM are provided in Table 1. The febrile group had a significantly higher proportion of cerebral cortical encephalitis (15/38 [39.5%] vs. 10/85 [11.8%]; *p* < 0.001). Compared to the afebrile group, the febrile group exhibited a significantly higher median CSF leukocyte count (56 [IQR, 20–172.8] vs. 11.5 [4–33] cells/μL; *p* < 0.001) and a greater proportion of patients with marked pleocytosis (> 50 cells/μL) (19/34 [55.9%] vs. 11/74 [14.9%]; *p* < 0.001). In parallel, the febrile group showed a significantly higher median CSF protein level (52.65 [IQR, 40.3–88.75] vs. 39.76 [30.06–48] mg/dL; *p* = 0.001) and a greater proportion of patients with protein elevation (> 45 mg/dL) (19/32 [59.4%] vs. 23/75 [30.7%]; *p* = 0.005). Furthermore, median CSF opening pressure was significantly elevated in the febrile group (190 [IQR, 158–243] vs. 160 [125–200] mmH_2_O; *p* = 0.015). Serum IL‐6 was available in 87 patients and showed no difference between febrile and afebrile groups. CSF IL‐6 was available in 49 patients; among these, a higher proportion of febrile patients had elevated CSF IL‐6 compared with afebrile patients (6/11 [54.5%] vs. 6/38 [15.8%]; *p* = 0.009). There were no statistically significant differences between the two groups in terms of disease duration (27 [IQR, 15.75–49.75] vs. 26 [11.5–44.5] month; *p* = 0.596) or relapsing course (23/38 [60.5%] vs. 47/85 [55.3%]; *p* = 0.588). In sensitivity analyses, we redefined fever using an axillary temperature cutoff of ≥ 38.1°C instead of ≥ 37.3°C, and the results remained consistent. After sensitivity analyses, the proportion of cerebral cortical encephalitis remained higher in the febrile group, though the difference was not statistically significant (8/23 [34.8%] vs. 17/100 [17%]; *p* = 0.056). Furthermore, the febrile group still exhibited significantly higher values in lumbar puncture opening pressure (190 [IQR, 150–250] vs. 162.5 [126.3–200] mmH_2_O; *p* = 0.035), CSF leukocyte (101.5 [IQR, 25.25–242.5] vs. 14.5 [4–41.5] cells/μL; *p* < 0.001), CSF protein level (52.65 [IQR, 40.92–86.47] vs. 40.2 [30.06–51.3] mg/dL; *p* = 0.01), and CSF IL‐6 level (274 [IQR, 5.4–756.3] vs. 6.96 [3.08–21.6] pg/mL; *p* = 0.037), as well as significantly higher proportions of elevated values for these parameters, compared with the afebrile group.

**TABLE 1:** Characteristics of patients with MOGAD with normal and high body temperature on attack.

	Full cohort (*n* = 123)	Febrile group^a^ (*n* = 38)	Afebrile group (*n* = 85)	*p*	Febrile group (> 38°C)^a^ (*n* = 23)	Afebrile group (≤ 38°C) (*n* = 100)	*p*
Age at onset, median (IQR), *y*	30 (22–42)	27.5 (18–42)	32 (24–41.50)	0.159	28 (21–42)	32 (23.25–41)	0.621
Female sex, No. (%)	64 (52.0)	17 (44.7)	47 (55.3)	0.279	11 (47.8)	53 (53)	0.654
Type of first MOGAD attack, No. (%)							
Optic neuritis (±brain lesion)	33 (26.8)	8 (21.1)	25 (29.4)	0.334	4 (17.4)	29 (29)	0.257
Myelitis	24 (19.5)	4 (10.5)	20 (23.5)	0.093	3 (13.0)	21 (21)	0.385
Both optic neuritis and myelitis	8 (6.5)	2 (5.3)	6 (7.1)	0.709	2 (8.7)	6 (6)	0.636
ADEM	4 (3.3)	3 (7.9)	1 (1.2)	0.052	2 (8.7)	2 (2)	0.103
Cerebral monofocal or polyfocal deficits	10 (8.1)	3 (7.9)	7 (8.2)	0.949	2 (8.7)	8 (8)	0.912
Cerebral cortical encephalitis	25 (20.3)	15 (39.5)	10 (11.8)	< 0.001*	8 (34.8)	17 (17)	0.056
Brainstem or cerebellar deficits	10 (8.1)	2 (5.3)	8 (9.4)	0.437	1 (4.4)	9 (9)	0.462
Other^b^	9 (7.3)	1 (2.6)	8 (9.4)	0.182	1 (4.4)	8 (8)	0.544
Severity at first MOGAD attack							
Worst EDSS at first attack, median (IQR)	3.0 (2.0–4.0)	3.0 (2.0–4.0)	3.0 (2.0–4.0)	0.357	2.0 (2.0–4.0)	3.0 (2.0–4.0)	0.249
EDSS ≥ 4, No. (%)	45/120 (37.5)	10/38 (26.3)	35/82 (42.7)	0.085	6/23 (26.1)	39/97 (40.2)	0.209
Laboratory tests							
Serum MOG‐IgG titer, median (IQR)	1:32 (1:10–1:100)	1:100 (1:10–1:155)	1:32 (1:10–1:100)	0.690	1:100 (1:32–1:320)	1:32 (1:10–1:100)	0.344
Serum MOG‐IgG ≥ 1:100	59/122 (48.4)	20/38 (52.6)	39/84 (46.4)	0.526	13/23 (56.5)	46/99 (46.5)	0.385
Serum IL‐6, median (IQR),^c^ pg/mL	2.45 (2–4.96)	3.69 (2–12)	2.20 (2–3.80)	0.057	3.09 (2–12.25)	2.34 (2–4.14)	0.125
Opening pressure, median (IQR), mmH_2_O	170 (130–220)	190 (158–243)	160 (125–200)	0.015*	190 (150–250)	162.5 (126.30–200)	0.035*
Raised OP,^d^ No. (%)	12/99 (12.1)	7/30 (23.3)	5/69 (7.2)	0.024*	5/19 (26.3)	7/80 (8.8)	0.035*
CSF leukocyte, median (IQR), cells/μL	20 (5–54.50)	56 (20–172.80)	11.50 (4–33)	< 0.001*	101.5 (25.25–242.50)	14.5 (4–41.50)	< 0.001*
CSF leukocyte > 50 cells/μL, No. (%)	30/108 (27.8)	19/34 (55.9)	11/74 (14.9)	< 0.001*	12/20 (60)	18/88 (20.5)	< 0.001*
CSF protein, median (IQR), mg/dL	41 (30.63–53.44)	52.65 (40.30–88.75)	39.76 (30.06–48)	0.001*	52.65 (40.92–86.47)	40.2 (30.06–51.30)	0.01*
CSF protein > 45 mg/dL, No. (%)	42/107 (39.3)	19/32 (59.4)	23/75 (30.7)	0.005*	12/20 (60)	30/87 (34.5)	0.035*
CSF‐unique OCB, No. (%)	27/87 (31)	9/24 (37.5)	18/63 (28.6)	0.421	4/16 (25)	23/71 (32.4)	0.564
CSF IL‐6, median (IQR), pg/mL	8.14 (3.27–34.85)	164 (3.43–487)	7.55 (3.09–21.40)	0.097	274 (5.40–756.30)	6.96 (3.08–21.60)	0.037*
CSF IL‐6 > 34 pg/mL,^e^ No. (%)	12/49 (24.5)	6/11 (54.5)	6/38 (15.8)	0.009*	5/8 (62.5)	7/41 (17.1)	0.006*
Meningeal enhancement on MRI, No. (%)	11/99 (11.1)	5/32 (15.6)	6/67 (9.0)	0.323	2/18 (11.1)	9/81 (11.1)	> 0.999
Post‐onset immunosuppressive therapy, No. (%)	39 (31.7)	13 (34.2)	26 (30.6)	0.69	9 (39.1)	30 (30)	0.40
Relapsing, No. (%)	70 (56.9)	23 (60.5)	47 (55.3)	0.588	14 (60.9)	56 (56)	0.671
Disease duration, median (IQR), *m*	27 (13–46)	27 (15.75–49.75)	26 (11.50–44.50)	0.596	22 (15–36)	27.50 (12–46.75)	0.628

Abbreviations: ADEM, acute disseminated encephalomyelitis; CSF, cerebrospinal fluid; EDSS, Kurtzke expanded disability status scale; IL‐6, interleukin‐6; MOGAD, myelin oligodendrocyte glycoprotein antibody‐associated disease; MOG‐IgG, anti‐myelin oligodendrocyte glycoprotein immunoglobulin G; OCB, oligoclonal band; OP, opening pressure.

^a^ Body temperature in febrile group is defined by 37.3°C or higher. A sensitivity analysis was performed for body temperature cutoff of 38.1°C or higher.

^b^ Other attack phenotypes include other mixed situations: cerebral monofocal or polyfocal deficits and brainstem or cerebellar deficits (7); brainstem or cerebellar deficits and myelitis (1); cerebral monofocal or polyfocal deficits and myelitis (1).

^c^ Eighty‐seven cases with available data, 23 in febrile group and 64 in afebrile group.

^d^ Raised OP is defined by 250 mmH_2_O or higher.

^e^ Thirty‐four is 10 times the upper limit of the normal range.

* *p* value is significant.

### 3.2 Clinical Feature of MOGAD Accompanied by Fever

Among the 38 patients in the febrile group, there were a total of 43 attacks accompanied by fever, 33 of which were the first attack. The detailed information of attacks accompanied by fever is presented in Table 2. The median maximum temperature during these episodes was 38.6°C (IQR, 38.0°C–39.28°C). Fever was classified as low‐grade (37.3°C–38.0°C) in 14 of 43 attacks (32.6%), moderate‐grade (38.1°C–39.0°C) in 14 (32.6%), high‐grade (39.1°C–41.0°C) in 9 (20.9%), ultrahyperpyrexia (≥ 41.1°C) in 2 (4.7%). One patient (2.3%) presented with ultrahyperpyrexia attributed to hypothalamic involvement. Regarding the temporal relationship between fever and neurological symptom onset across the 43 febrile episodes, fever occurred 1–3 days before symptom onset in 3 episodes (7.0%), 0–3 days after symptom onset in 34 episodes (79.1%), and 4–7 days after symptom onset in 6 episodes (14.0%). Among the 43 febrile episodes, mild cold‐like prodromes were noted in 2 cases, pneumonia in 1, and cold exposure in 1; no patient reported recent vaccination. The clinical phenotype associated with febrile attacks was diverse. Cerebral cortical encephalitis was the most common phenotype among febrile attacks. Of the 43 febrile attacks, 21 (48.8%) presented with cerebral cortical encephalitis, 8 (18.6%) with myelitis, 10 (23.3%) with cerebral monofocal or polyfocal deficits, 8 (18.6%) with optic neuritis (1 of which was isolated optic neuritis), 3 (7%) with ADEM, and 4 (9.3%) with brainstem or cerebellar deficits. Of the 43 febrile attacks, 33 were confirmed positive for serum MOG‐IgG via CBA at the time of the attack; in one additional case, a tissue‐based assay was positive, though the target antigen could not be identified at that time. Of the 43 febrile attacks, pathogen next‐generation sequencing (NGS) was available for 20 attacks and bacterial culture for 13 attacks. Only 2 patients' CSF NGS revealed suspicious positive results with extremely low read counts (3 reads for human herpesvirus 4 and 1 read for human herpesvirus 1). However, both attacks were accompanied by concurrent serum MOG antibody positivity (with titers of 1:32 and 1:320, respectively).

**TABLE 2:** Clinical features of MOGAD attack with fever.

Attack of MOGAD with fever, descriptive characteristics (*N* = 43)	
Peak body temperature,^a^ median (IQR), °C	38.6 (38.0–39.28)
Fever grade, No. (%)	
Low‐grade (37.3°C–38°C)	14 (32.6)
Moderate‐grade (38.1°C–39°C)	14 (32.6)
High‐grade (39.1°C–41°C)	9 (20.9)
Ultrahyperpyrexia (≥ 41.1°C)	2 (4.7)
Fever occurring time, No. (%)	
1–3 day before symptom onset	3 (7)
0–3 days after symptom onset	34 (79.1)
4–7 days after symptom onset	6 (14)
Phenotype of febrile attack, No. (%)	
Optic neuritis	8 (18.6)
Myelitis	8 (18.6)
ADEM	3 (7.0)
Cerebral monofocal or polyfocal deficits	10 (23.3)
Brainstem or cerebellar deficits	4 (9.3)
Cerebral cortical encephalitis	21 (48.8)
Mix^b^	9 (20.9)
Episode type, No. (%)	
First attack	33 (76.7)
Relapse	10 (23.3)
Laboratory features	
MOG‐IgG positivity before or during the attack	33/43 (76.7)
NGS‐negative attack, No. (%)	18/20 (90)
Blood CRP, median, mg/L	3.51 (1.73–8.34)
Blood CRP > 10 mg/L, No. (%)	4/20 (20)
Blood leukocyte, median (IQR), cells/μL	9.35 × 10^3^ (7.13 × 10^3^–13.34 × 10^3^)
Blood leukocyte > 10 × 10^3^/μL, No. (%)	14/29 (48.3)

Abbreviations: ADEM, acute disseminated encephalomyelitis; CRP, C‐reactive protein; MOG, myelin oligodendrocyte glycoprotein; MOG‐IgG, anti‐myelin oligodendrocyte glycoprotein immunoglobulin G; NGS, next‐generation sequencing; WBC, white blood cell.

^a^ Temperature data were recorded for 38 attacks (one additional attack was documented as low‐grade fever only, and four attacks had missing data).

^b^ Mix situations include: optic neuritis and cerebral cortical encephalitis (2), optic neuritis and myelitis (2), optic neuritis and cerebral monofocal or polyfocal deficits (1), optic neuritis, cerebral monofocal or polyfocal deficits and brainstem or cerebellar deficits (2); brainstem or cerebellar deficits and myelitis (1); cerebral monofocal or polyfocal deficits and myelitis (1).

Empirical antimicrobial therapy was administered in 81.4% of febrile attacks (35/43), and first‐line immunotherapies were initiated in 90.7% (39/43). Immediate defervescence and symptomatic improvement following corticosteroid or intravenous immunoglobulin administration were observed in 11 febrile attacks.

### 3.3 Factors Associated With Fever and Relapse Outcomes in MOGAD Patients

We first screened potential clinical factors for fever using univariate logistic regression, followed by multivariable analysis to adjust for confounders (Table 3). The univariate logistic analysis of all clinical factors showed that cerebral cortical encephalitis (OR, 8.00; 95% CI, 1.84–34.79; *p* = 0.006), elevated CSF opening pressure (OR, 3.9; 95% CI, 1.12–13.50; *p* = 0.032), CSF leukocyte > 50 cells/μL (OR, 7.25; 95% CI, 2.86–18.42; *p* < 0.01) and CSF protein > 45 mg/dL (OR, 3.30; 95% CI, 1.40–7.8; *p* = 0.006) were factors associated with fever in patients with MOGAD. In our multivariable binary logistic analysis, the likelihood of fever was 7.01‐fold higher (95% CI, 1.83–26.91; *p* = 0.005) in the CSF leukocyte > 50 cells/μL group than in the CSF leukocyte ≤ 50 cells/μL group. In the cerebral cortical encephalitis group, there was a trend to increased rate of patients with fever compared with the ON group (OR, 8.87; 95% CI, 1.41–55.59; *p* = 0.020). Moreover, lumbar pressure (OR, 0.8; 95% CI, 0.14–4.39; *p* = 0.793), and CSF protein (OR, 2.91; 95% CI, 0.84–10.15; *p* = 0.093) were not independently associated with fever in the multivariable model.

**TABLE 3:** Univariate and multivariate logistic regression results for predictors of fever.

	Univariate analysis					Multivariate analysis				
	*β*	SE	*Z*	*p*	OR (95% CI)	*β*	SE	*Z*	*p*	OR (95% CI)
Age at onset										
< 45					1.00 (Reference)					
≥ 45	−0.43	0.55	−0.77	0.443	0.65 (0.22–1.94)					
Sex										
Female					1.00 (Reference)					
Male	0.42	0.39	1.08	0.280	1.53 (0.71–3.30)					
Phenotype										
Optic neuritis					1.00 (Reference)					1.00 (Reference)
Myelitis	0.06	0.83	0.08	0.938	1.07 (0.21–5.47)	−0.18	1.02	−0.18	0.857	0.83 (0.11–6.12)
Cerebral cortical encephalitis	2.08	0.75	2.77	0.006^a^	8.00 (1.84–34.79)	2.18	0.94	2.33	0.020^*^	8.87 (1.41–55.59)
Other^b^	0.78	0.70	1.13	0.260	2.19 (0.56–8.56)	0.52	0.86	0.61	0.544	1.69 (0.31–9.11)
CSF OP pressure										
OP < 250 mmH_2_O					1.00 (Reference)					1.00 (Reference)
OP ≥ 250 mmH_2_O	1.36	0.63	2.14	0.032^a^	3.90 (1.12–13.50)	−0.23	0.87	−0.26	0.793	0.80 (0.14–4.39)
CSF leukocyte										
Leukocyte ≤ 50 cells/μL					1.00 (Reference)					1.00 (Reference)
Leukocyte > 50 cells/μL	1.98	0.48	4.17	< 0.001^a^	7.25 (2.86–18.42)	1.95	0.69	2.84	0.005^*^	7.01 (1.83–26.91)
CSF protein										
Protein ≤ 45 mg/dL					1.00 (Reference)					1.00 (Reference)
Protein > 45 mg/dL	1.20	0.44	2.73	0.006^a^	3.30 (1.40–7.80)	1.07	0.64	1.68	0.093	2.91 (0.84–10.15)
Meningeal enhancement										
Absent					1.00 (Reference)					
Present	0.63	0.65	0.98	0.329	1.88 (0.53–6.71)					
Serum MOG‐ IgG titer										
MOG‐IgG titer < 1:100					1.00 (Reference)					
MOG‐IgG titer ≥ 1:100	0.27	0.39	0.69	0.489	1.31 (0.61–2.82)					
Relapsing course										
Absent					1.00 (Reference)					
Present	0.21	0.40	0.54	0.588	1.24 (0.57–2.70)					

Abbreviations: CI, confidence interval; CSF, cerebrospinal fluid; MOG‐IgG, anti‐myelin oligodendrocyte glycoprotein immunoglobulin G; OP, opening pressure; OR, odds ratio; SE, standard error.

^a^ Candidate predictors (*p* < 0.2) in the univariate analysis.

^b^ Other phenotypes include acute disseminated encephalomyelitis, cerebral monofocal or polyfocal deficits, cerebral cortical encephalitis, brainstem or cerebellar deficits, and other mixed situations.

* *p* < 0.05 in the multivariate analysis.

**FIGURE 2:**
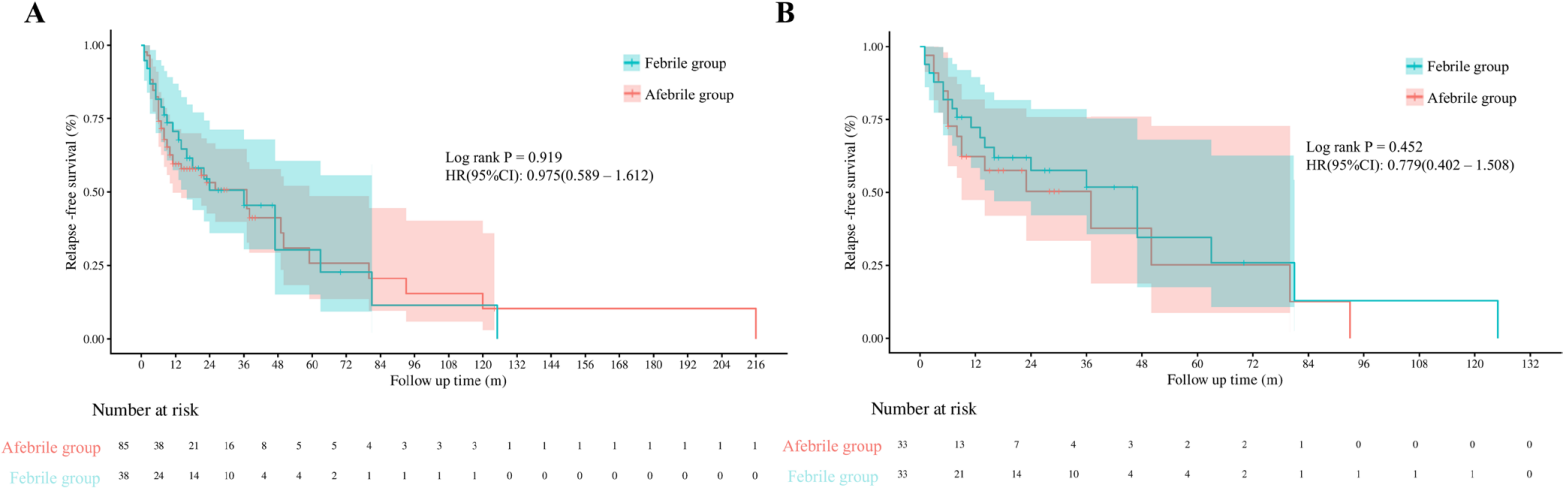
Kaplan–Meier survival analysis. (A) Kaplan–Meier analysis of time to first relapse between febrile and afebrile group before post‐propensity score matching (PSM). No significant difference was observed between the two groups (Log‐rank *p* = 0.919). (B) Kaplan–Meier analysis of time to first relapse after PSM. No significant difference was observed between the two groups (Log‐rank *p* = 0.452).

To determine whether fever status holds prognostic value for disease relapse, we performed Kaplan–Meier survival analysis to compare the distribution of time‐to‐relapse between febrile and afebrile groups (Figure 2A). In the total cohort, Kaplan–Meier survival analysis revealed no statistically significant difference in the time to confirmed relapse between the 2 groups (Log‐rank *p* = 0.919).

### 3.4 Patient Characteristics and Relapse Outcomes in the Propensity‐Matched Cohort

Following PSM, which adjusted for a comprehensive set of covariates including age, gender, onset phenotype, EDSS score, serum MOG‐IgG titer, and onset immunosuppressive therapy, 33 well‐balanced pairs were selected (Table S1). In the matched cohort, absolute SMD for all covariates was reduced to below 0.2, indicating adequate balance. The baseline demographic and clinical characteristics of the propensity‐matched cohort are summarized in Table 4. Notably, CSF leukocyte and protein remained significantly higher between the febrile and afebrile groups even after matching. Specifically, patients in the febrile group had significantly higher median CSF leukocyte (55 [IQR, 20–173.5] vs. 13 [5–42.5] cells/μL; *p* = 0.002), median CSF protein (52.5 [IQR, 38.99–90] vs. 40.2 [29.14–48.15] mg/dL; *p* = 0.025), proportion of CSF pleocytosis (16/29 [55.2%] vs. 4/29 [13.8%]; *p* < 0.001), and proportion of elevated CSF protein (16/27 [59.3%] vs. 7/29 [24.1%]; *p* = 0.008). Kaplan–Meier analysis of the matched pairs demonstrated no significant difference in the risk of relapse between the two groups (log‐rank *p* = 0.452) (Figure 2B).

**TABLE 4:** Characteristics of patients with MOGAD after PSM.

	Full cohort (*n* = 66)	Febrile group (*n* = 33)	Afebrile group (*n* = 33)	*p*
Age at onset, median (IQR), *y*	29.5 (21.75–39.50)	29 (18.50–42)	32 (25.5–38)	0.581
Female sex, No. (%)	29 (43.9)	16 (48.5)	13 (39.4)	0.457
Type of first MOGAD attack, No. (%)				
Optic neuritis (±brain lesion)	15 (22.7)	8 (24.2)	7 (21.2)	0.769
Myelitis	8 (12.1)	4 (12.1)	4 (12.1)	> 0.999
Both optic neuritis and myelitis	3 (9.1)	2 (6.1)	1 (3.0)	0.555
ADEM	3 (4.5)	3 (9.1)	0 (0)	0.08
Cerebral monofocal or polyfocal deficits	8 (12.1)	3 (9.1)	5 (15.2)	0.451
Cerebral cortical encephalitis	20 (30.3)	10 (30.3)	10 (30.3)	> 0.999
Brainstem or cerebellar deficits	4 (6.1)	2 (6.1)	2 (6.1)	> 0.999
Other^a^	5 (15.2)	1 (3.0)	4 (12.1)	0.163
Severity at first MOGAD attack				
Worst EDSS at first attack, median (IQR)	2.0 (2.0–4.0)	3.0 (2.0–4.0)	2.0 (2.0–4.0)	0.674
EDSS ≥ 4, No. (%)	20 (30.3)	10 (30.3)	10 (30.3)	> 0.999
Laboratory tests				
Serum MOG‐IgG titer, median (IQR)	1:100 (1:32–1:320)	1:100 (1:21–1:210)	1:100 (1:32–1:320)	0.821
Serum MOG‐IgG ≥ 1:100	35/65 (53.8)	18/33 (54.6)	17/32 (53.1)	0.909
Serum IL‐6, median (IQR),^b^ pg/mL	2.52 (2–5.67)	3.15 (2–8.85)	2.34 (2–5.0)	0.366
Opening pressure, median (IQR), mmH_2_O	190 (150–232.5)	195 (157.5–242.5)	185 (146.3–223.8)	0.264
Raised OP, No. (%)	9/54 (16.7)	6/26 (23.1)	3/28 (10.7)	0.223
CSF leukocyte, median (IQR), cells/μL	29.5 (6–120.3)	55 (20–173.5)	13 (5–42.5)	0.002^*^
CSF leukocyte > 50 cells/μL, No. (%)	20/58 (34.5)	16/29 (55.2)	4/29 (13.8)	< 0.001^*^
CSF protein, median (IQR), mg/dL	41.97 (31.66–61.62)	52.5 (38.99–90)	40.2 (29.14–48.15)	0.025^*^
CSF protein > 45 mg/dL, No. (%)	23/56 (41.1)	16/27 (59.3)	7/29 (24.1)	0.008^*^
CSF‐unique OCB, No. (%)	16/44 (36.4)	8/21 (38.1)	8/23 (34.8)	0.820
CSF IL‐6, median (IQR), pg/mL	5.99 (3.27–228)	179 (3.26–576.8)	5.54 (3.1–21.2)	0.290
CSF IL‐6 > 34 pg/mL, No. (%)	7/21 (33.3)	5/10 (50)	2/11 (18.2)	0.122
Meningeal enhancement on MRI, No. (%)	5/56 (8.9)	2/27 (7.4)	3/29 (10.3)	0.700
Post‐onset immunosuppressive therapy	20 (30.3)	11 (33.3)	9 (27.3)	0.592
Relapsing, No. (%)	37 (56.1)	19 (57.6)	18 (54.6)	0.804
Disease duration, median (IQR), *m*	27.5 (10.75–49.25)	28 (17–52.5)	22 (9–49.5)	0.195

Abbreviations: EDSS, expanded disability status scale; IS, immunosuppressive therapy; MOG‐IgG, anti‐myelin oligodendrocyte glycoprotein immunoglobulin G; PSM, propensity score matching.

^a^ Other phenotypes include acute disseminated encephalomyelitis, cerebral monofocal or polyfocal deficits, cerebral cortical encephalitis, brainstem or cerebellar deficits, and other mixed situations.

^b^ Forty‐two cases with available data, 21 in febrile group and 21 in afebrile group.

* *p* value is significant.

## 4 Discussion

In this cohort, 30.9% of patients with MOGAD presented with fever during acute attacks, with maximal temperatures predominantly below 39°C. Cerebral cortical encephalitis was the most frequent phenotype in the febrile group. Independent predictors of fever included cerebral cortical encephalitis and CSF pleocytosis. Moreover, febrile patients had a significantly higher prevalence of elevated CSF opening pressure, CSF protein, CSF leukocyte count, and CSF IL‐6 levels.

Previous literature has mentioned the fever rate in MOGAD was 9%–39% [7, 15]. This ratio was higher in MOGAD encephalitis cases. The overall frequency of fever in MOGAD encephalitis is 55%–75% [10, 16, 17], yet it is notably higher (66.7%) among patients with cortical encephalitis [18], a finding that aligns with our results identifying cortical encephalitis as the predominant phenotype. Although fever has been reported to be uniformly absent in isolated optic neuritis in prior case series [7], our cohort included one case of isolated optic neuritis accompanied by fever. Of note, another such case was lost to follow‐up and therefore excluded from the final cohort. Fever was observed across all MOGAD phenotypes, suggesting it is a disease‐inherent feature rather than phenotype‐specific. Fever independent of central hyperpyrexia in neuromyelitis optica spectrum disorder (NMOSD) has been rarely reported [19, 20]. In MS, fever is a red flag for relapse [4]. Furthermore, fever can also occur in autoimmune glial fibrillary acidic protein astrocytopathy (GFAP‐A) patients [20]. Both GFAP‐A and MOGAD are great mimickers that can present with CNS infection‐like manifestations at onset, suggesting a potential overlap in their underlying pathogenic mechanisms. The high frequency of fever in MOGAD argues against its role as a warning sign and instead points to it as a distinctive feature that sets the disease apart from NMOSD and MS. Fever, particularly when accompanied by CSF pleocytosis, poses a diagnostic pitfall in CNS autoimmune disorders, where it is traditionally viewed as rare. This deceptive presentation frequently biases clinicians toward infectious differentials, such as viral encephalitis or acute flaccid myelitis, with these conditions often being the initial diagnosis in a considerable number of cases, diverting attention from MOGAD and thereby postponing MOG‐IgG testing and definitive diagnosis [9, 21, 22, 23]. And early intervention during the first acute MOGAD attack correlated with a lower relapse rate and a higher chance of MOG‐IgG seronegativity [24]. Therefore, recognizing that MOGAD can present with fever is essential to refine diagnostic thinking and prompt earlier antibody testing.

We consider two possible mechanisms by which fever may occur in MOGAD. On one hand, IL ‐ 6, an endogenous pyrogen [25], profoundly elevated in CSF. On the other hand, inflammatory lesion involved hypothalamus. But they are unlikely to provide a complete explanation for fever in MOGAD. In MOGAD, CSF IL‐6 level was markedly higher than that in serum samples, and this elevation showed a positive correlation with CSF pleocytosis [26]. In our study, CSF leukocyte was higher in the febrile group than in the afebrile group. CSF pleocytosis, in conjunction with elevated IL‐6 levels, may reflect a more intense central nervous system inflammatory state in febrile patients. However, there was no significant difference between two groups in relapse‐free rate following the first attack. This finding echoes a previous report in MS showing that CSF pleocytosis does not portend a worse long‐term outcome [27]. Fever, CSF pleocytosis, elevated CSF opening pressure, and increased protein levels are classic signs suggestive of CNS infection. Moreover, MOGAD is well‐documented as a post‐ or para‐infectious condition [28, 29, 30, 31]. Nevertheless, several lines of evidence suggest that fever in our MOGAD cohort may not be entirely attributable to infection. First, a narrow time window between fever onset and neurological symptoms was deliberately applied to minimize the likelihood of a pre‐existing infection trigger [32]. Second, none of the patients had a recent history of vaccination, and only a minority of attacks had documented evidence of systemic infectious events, which were mostly mild in nature. Third, more than half of the febrile patients in our cohort had negative pathogenic evidence, as confirmed by CSF NGS and bacterial cultures. And neither peripheral leukocyte counts nor CRP level was markedly elevated. Fourth, a subset of patients showed a favorable response to corticosteroid therapy [12]. Collectively, these findings argue against infection as the sole driver of fever in the majority of our febrile patients, and raise the possibility that fever is, at least in part, intrinsic to MOGAD biology. In our cohort, febrile episodes occurred predominantly at first attack but were far less frequent during relapses, a difference that may be partly attributable to oral corticosteroid use [33]. Biomarkers that distinguish infection from MOGAD reactivation would be of clinical value and warrant further investigation.

This study has several limitations. First, this is a retrospective study with limitations typical of a retrospective study design. Second, detailed information, such as fever duration and fever pattern, was seldom recorded. Third, we did not compare the frequency of fever with that in other neuroinflammatory disorders. Further studies are needed to elucidate the precise mechanisms of fever in MOGAD.

Our large cohort data show that fever occurred in a substantial proportion (30.9%) of patients with MOGAD, after excluding those with prodromal fever. Whether attributable to the disease process or to a concomitant infection, fever occurs alongside the disease presentation. Thus, fever, even when accompanied by prominent CSF pleocytosis, should not be used to exclude MOGAD; rather, it should be recognized as a potential component of the initial clinical picture.

## Author Contributions

Concept and design: K. Shi. Drafting of the manuscript: A. Guo. Intellectual content: F.‐D. Shi. Statistical analysis: A. Guo. Study supervision: F.‐D. Shi and K. Shi. Acquisition, analysis, or interpretation of data: all authors.

## Funding

The authors have nothing to report.

## Ethics Statement

This study was approved by the Ethics Committee of Beijing Tiantan Hospital (approval ID: KY2021‐150‐02) in accordance with the 1964 Declaration of Helsinki and its later amendments. Written informed consent was obtained from all patients or their legal representative.

## Conflicts of Interest

The authors declare no conflicts of interest.

## Supporting information


**Table S1:** Comparison of baseline characteristics between febrile and afebrile patients with MOGAD before and after PSM.

## Data Availability

The data that support the findings of this study are available from the corresponding author upon reasonable request.
