# Supplementary material for: Frequency and Characteristics of Myelin Oligodendrocyte Glycoprotein Antibody‐Associated Disease Accompanied by Fever: A Single‐Center Retrospective Study
Source: CNS Neurosci Ther. 2026 Sep 15;32(9):e71153. doi: 10.1002/cns.71153 (PMC13576922; doi:10.1002/cns.71153)
Supplement: Supplementary file 1 [file CNS-32-e71153-s001.docx]

| Variable  Table S1. Comparison of baseline characteristics between febrile and afebrile patients with MOGAD before and after PSM | Before PSM | | | | | |  | After PSM | | | | | |
| --- | --- | --- | --- | --- | --- | --- | --- | --- | --- | --- | --- | --- | --- |
|  | Total (n = 123) | Afebrile group  (n = 85) | Febrile group  (n = 38) | Statistic | *P* | SMD |  | Total (n = 66) | Afebrile group  (n = 33) | Febrile group  (n = 33) | Statistic | *P* | SMD |
| Age at onset, median (IQR), y | 30.00 (22.50, 41.50) | 32.00 (24.00, 41.00) | 27.50 (18.25, 41.75) | Z=-1.410 | 0.158 | -0.310 |  | 29.50 (22.00, 38.75) | 32.00 (26.00, 38.00) | 29.00 (19.00, 42.00) | Z=-0.552 | 0.581 | -0.097 |
| Gender, n (%) |  |  |  | χ²=1.173 | 0.279 |  |  |  |  |  | χ²=0.554 | 0.457 |  |
| Male | 59 (47.97) | 38 (44.71) | 21 (55.26) |  |  | 0.212 |  | 37 (56.06) | 20 (60.61) | 17 (51.52) |  |  | -0.182 |
| Female | 64 (52.03) | 47 (55.29) | 17 (44.74) |  |  | -0.212 |  | 29 (43.94) | 13 (39.39) | 16 (48.48) |  |  | 0.182 |
| Phenotype, n (%) |  |  |  | χ²=14.310 | 0.003 |  |  |  |  |  | - | 1.000 |  |
| Optic neuritis | 19 (15.45) | 16 (18.82) | 3 (7.89) |  |  | -0.405 |  | 7 (10.61) | 4 (12.12) | 3 (9.09) |  |  | -0.105 |
| Myelitis | 24 (19.51) | 20 (23.53) | 4 (10.53) |  |  | -0.424 |  | 8 (12.12) | 4 (12.12) | 4 (12.12) |  |  | 0.000 |
| Cerebral cortical encephalitis | 25 (20.33) | 10 (11.76) | 15 (39.47) |  |  | 0.567 |  | 20 (30.3) | 10 (30.30) | 10 (30.30) |  |  | 0.000 |
| Others^a^ | 55 (44.72) | 39 (45.88) | 16 (42.11) |  |  | -0.077 |  | 31 (46.97) | 15 (45.45) | 16 (48.48) |  |  | 0.061 |
| EDSS, n (%) |  |  |  | χ²=2.968 | 0.085 |  |  |  |  |  | χ²=0.000 | 1.000 |  |
| EDSS <4, No. (%) | 75 (62.5) | 47 (57.32) | 28 (73.68) |  |  | 0.372 |  | 46 (69.7) | 23 (69.70) | 23 (69.70) |  |  | 0.000 |
| EDSS ≥4, No. (%) | 45 (37.5) | 35 (42.68) | 10 (26.32) |  |  | -0.372 |  | 20 (30.3) | 10 (30.30) | 10 (30.30) |  |  | 0.000 |
| Serum titer, n (%) |  |  |  | χ²=0.479 | 0.489 |  |  |  |  |  | χ²=0.061 | 0.805 |  |
| MOG-IgG <1:100 | 64 (52.03) | 46 (54.12) | 18 (47.37) |  |  | -0.135 |  | 31 (46.97) | 16 (48.48) | 15 (45.45) |  |  | -0.061 |
| MOG-IgG ≥ 1:100 | 59 (47.97) | 39 (45.88) | 20 (52.63) |  |  | 0.135 |  | 35 (53.03) | 17 (51.52) | 18 (54.55) |  |  | 0.061 |
| Therapy, n (%) |  |  |  | χ²=0.159 | 0.690 |  |  |  |  |  | χ²=0.287 | 0.592 |  |
| Without Post-onset IS therapy, n (%) | 84 (68.29) | 59 (69.41) | 25 (65.79) |  |  | -0.076 |  | 46 (69.7) | 24 (72.73) | 22 (66.67) |  |  | -0.129 |
| Post-onset IS  therapy, n (%) | 39 (31.71) | 26 (30.59) | 13 (34.21) |  |  | 0.076 |  | 20 (30.3) | 9 (27.27) | 11 (33.33) |  |  | 0.129 |

Abbreviations: PSM, propensity score matching; SMD, Standardized Mean Difference; EDSS, Expanded Disability Status Scale; MOG-IgG, anti-myelin oligodendrocyte glycoprotein immunoglobulin G; IS: immunosuppressive therapy

a. Other phenotypes include acute disseminated encephalomyelitis, cerebral monofocal or polyfocal deficits, cerebral cortical encephalitis, brainstem or cerebellar deficits and other mixed situations.
